# Esophageal cancer and precancerous lesions: focus on resident bacteria and fungi

**DOI:** 10.1128/spectrum.03137-24

**Published:** 2025-05-20

**Authors:** Liping Yuan, Yi Zhang, Chengli Wen, Sha Liu, Qin Zhang, Wen Yin, Qian Jia, Maolin Chen, Gang Luo, Mingming Deng, Muhan Lv, Wanmeng Xiao

**Affiliations:** 1 Department of Gastroenterology, The Affiliated Hospital, Southwest Medical University 666679, Luzhou, China; 2 Department of Gastroenterology, The First People’s Hospital of Liangshan Yi Autonomous Prefecture, Xichang, China; 3 Molecular Imaging and Therapy Research Unit, Department of Radiologic Technology, Faculty of Associated Medical Sciences, Chiang Mai University 626959, Chiang Mai, Thailand; 4 Department of Critical Care Medicine, The Affiliated Hospital, Southwest Medical University 691458, Luzhou, China; 5 Human Microecology and Precision Diagnosis and Treatment of Luzhou Key Laboratory, Luzhou, China; 6 Department of Pharmacy, The Affiliated Hospital, Southwest Medical University 74647, Luzhou, China; Tainan Hospital Ministry of Health and Welfare, Tainan, Taiwan

**Keywords:** esophageal squamous cell carcinoma, esophageal squamous intraepithelial neoplasia, bacteria, fungi, microbiome

## Abstract

Accumulating evidence highlights the pivotal role of microbiomes in cancer development. To elucidate the esophageal microbiome’s characteristics during esophageal squamous cell carcinoma (ESCC) progression, normal tissues from 13 healthy controls (HC), paired esophageal squamous intraepithelial neoplasia (ESIN) lesional and adjacent (ESINA) tissues from 10 ESIN individuals, and ESCC lesional and adjacent (ESCCA) tissues from 12 ESCC individuals were collected. Following 16S rRNA and ITS sequencing, analyses encompassed α/β-diversity assessments, shared species identification, Type-I Taylor’s Power Law Extensions (TPLE), linear discriminant analysis effect size (LEfSe), co-occurrence networks, receiver operating characteristic (ROC) curve analysis, and functional predictions. Distinct microbial signatures characterized HC, precancerous, and cancerous groups. The ESIN group exhibited unique microbial features, including diminished bacterial and fungal species sharing relative to the ESINA group and maximal *b* values in TPLE for both taxa. Despite the absence of significant bacterial composition differences between HC and ESIN in β-diversity analysis, notable alterations in the oral microbiome were observed. ESIN was marked by *Lactobacillus* and *Aspergillus* enrichment, while ESCC was predominantly associated with *Fusobacterium*, *Streptococcus*, and *Alternaria*. Disease progression led to shifts and reductions in species co-occurrence network interactions. *Aspergillus* demonstrated potential diagnostic value for ESIN, and its ratio to pathogenic functional clusters within network analysis significantly enhanced ESCC detection accuracy. Functional predictions revealed stage-specific pathway enrichments. These findings delineate microbiome alterations across ESCC stages, emphasizing ESIN-specific microbial shifts that may inform microbiome-based strategies for early detection and intervention.

**IMPORTANCE:**

This study collected esophageal tissues through gastroscopic biopsy and conducted sequencing and analyses to explore the diversity, heterogeneity, key microbial composition, interaction networks, and functional predictions of resident bacteria and fungi in esophageal squamous cell carcinoma (ESCC) progression. The esophageal squamous intraepithelial neoplasia group showed the highest heterogeneity in oral microbiome and fungi, with certain species potentially contributing to ESCC progression. Targeting the oral microbiome in high-risk populations may thus provide a valuable approach for improving early diagnosis and potentially intervening in disease progression.

## INTRODUCTION

Esophageal cancer is a prevalent malignancy and a major contributor to the global disease burden (1). In 2020, 604,100 new cases were reported worldwide (2). The two primary histological subtypes are esophageal squamous cell carcinoma (ESCC) and esophageal adenocarcinoma, with ESCC exhibiting a particularly high incidence in China, accounting for over half of global cases (3). The majority of patients with ESCC are diagnosed at advanced stages, with a 5-year survival rate of only 30.3% (4). Esophageal squamous intraepithelial neoplasia (ESIN) represents a precancerous stage in ESCC tumorigenesis (5), with longitudinal studies indicating that 31–74% of ESIN cases progress to ESCC over time (6). Although histopathological criteria for malignant epithelial transformations are well established, the molecular and microbial mechanisms driving ESIN-to-ESCC progression remain poorly understood.

The human digestive tract harbors a vast and complex microbiome that interacts dynamically with the host to maintain homeostasis. Microbial dysbiosis, characterized by reduced diversity and enrichment of pathogenic taxa, has been implicated in the pathogenesis of various diseases, including cancer (7). Increasing evidence suggests that the microbiome is a distinguishing feature of malignancies. A recent study revealed significant differences in the microbial composition of saliva and esophageal swabs between ESIN/ESCC patients and control individuals (8). However, these sampling strategies primarily capture the transient microbiome rather than the resident esophageal microbiome. Endoscopic biopsy studies have provided deeper insights into esophageal microbial composition. For instance, Shao et al. reported decreased microbial diversity in ESCC compared to the healthy controls (HC) group, with a notable increase in *Fusobacterium* (9). Similarly, Jiang et al. observed an enrichment of *Streptococcus* spp. and a depletion of *Faecalibacterium*, *Bacteroides*, and *Curvibacter* in ESCC relative to esophagitis (10). However, these studies focused exclusively on bacterial communities, overlooking fungi, which constitute a crucial yet underexplored component of the esophageal microbiome (11–13). Despite growing interest in the role of fungi in oncogenesis, research on fungal communities in esophageal cancer remains scarce.

This study aimed to comprehensively characterize the esophageal microbiome, encompassing both bacterial and fungal communities, across different stages of ESCC progression. Using gastroscopic biopsy specimens from five groups—HC, esophageal squamous intraepithelial neoplasia adjacent (ESINA) tissues, ESIN, esophageal squamous cell carcinoma adjacent (ESCCA) tissues, and ESCC—sequencing and bioinformatics analyses were performed to assess microbial diversity, heterogeneity, key taxa, interaction networks, and functional predictions. The findings may provide novel insights into microbiome alterations and the potential involvement of pathogenic microorganisms in ESCC development.

## MATERIALS AND METHODS

### Study group and sample collection

**Fig 1:**
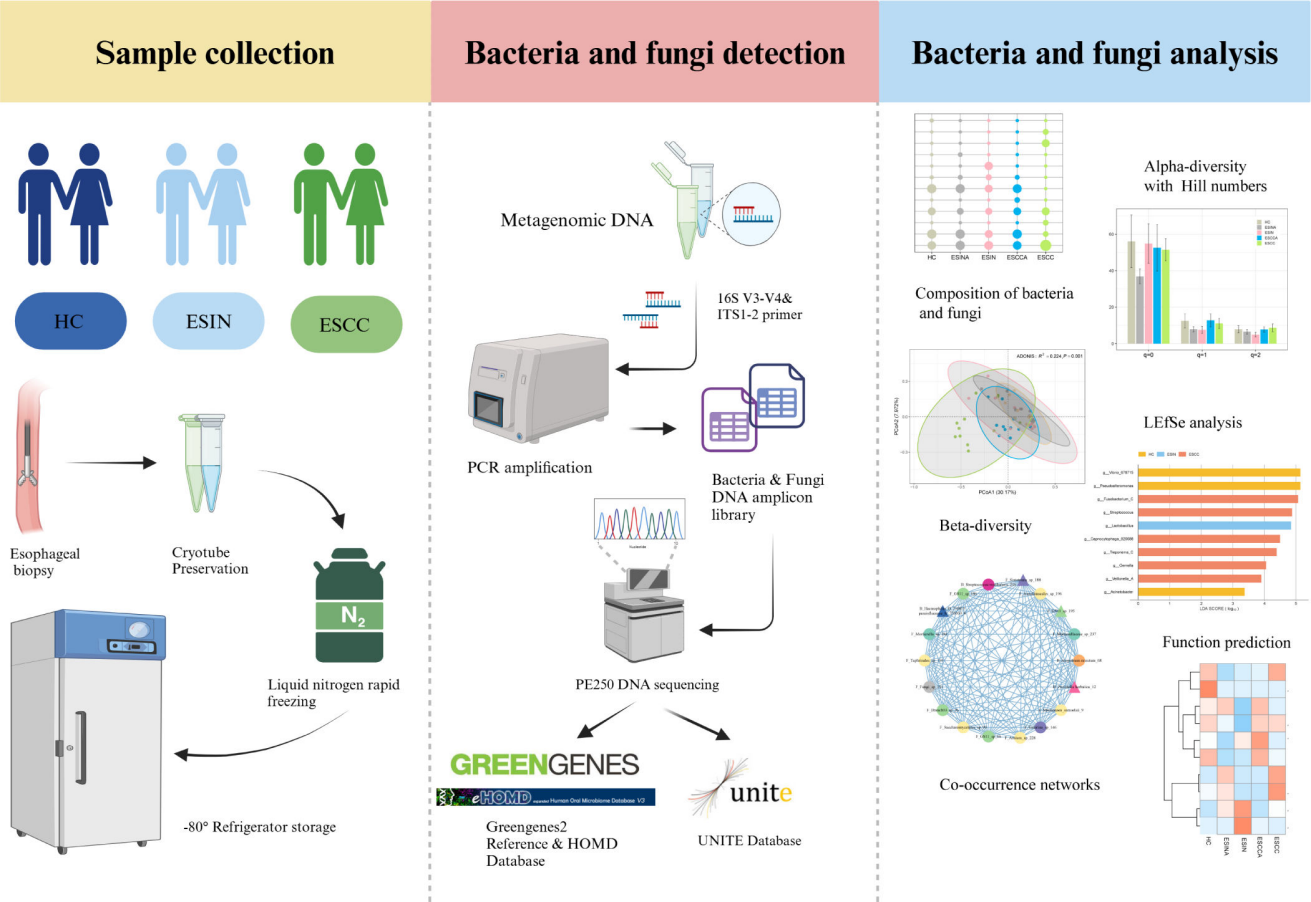
Schematic overview of the study design and objectives. ESCC, esophageal squamous cell carcinoma; ESIN, esophageal squamous intraepithelial neoplasia; HC, healthy controls; ITS, internal transcribed spacer; LEfSe, linear discriminant analysis effect size.

A case-control study was conducted at the Department of Gastroenterology, Affiliated Hospital of Southwest Medical University, Luzhou, from July 2023 to August 2024. The overall study design and objectives are illustrated in Fig. 1. The cohort included 13 HC individuals with no endoscopic evidence of esophageal disease, 10 patients with histopathologically confirmed ESIN, and 12 patients with ESCC. Participant selection adhered to the following inclusion criteria: (i) histopathological confirmation of ESIN, ESCC, or absence of endoscopic abnormalities; (ii) no prior history of malignancies or anticancer treatments; and (iii) willingness to provide esophageal biopsy samples for research purposes. Exclusion criteria encompassed: (i) individuals under 18 years of age; (ii) recent pharmacotherapy, including oral, intramuscular, or intravenous antibacterial drugs, probiotics, or other microbiome-altering medications within the past month; and (iii) presence of metastatic malignancies or recurrent ESCC. Written informed consent was obtained from all participants before enrollment. Clinical and demographic data, including age, smoking and alcohol consumption status, dietary habits, annual income, and family medical history, were collected through structured questionnaires administered by trained personnel.

Sterile biopsy forceps were used to collect paired esophageal tissues during gastroscopy or endoscopic submucosal dissection (ESD), comprising ESCC and ESCCA, ESIN, and ESINA, as well as esophageal tissue from HC individuals. The sampling workflow and quality control measures are detailed in the Supplemental data. Immediately following collection, esophageal tissues were snap-frozen in liquid nitrogen and stored at –80°C for subsequent analysis.

### DNA extraction, 16S rRNA, and internal transcribed spacer (ITS) sequencing

Genomic DNA was extracted using the Bacterial DNA Extraction Kit following the manufacturer’s protocol. Purity and concentration were assessed with a Nanodrop One spectrophotometer (Thermo Fisher Scientific, MA, USA). PCR amplification targeted the 16S rRNA V3–V4 region using primers 338F (ACTCCTACGGGAGGCAGCA) and 806R (GGACTACHVGGGTWTCTAAT), while fungal ITS regions were amplified with BD-ITS1F (CTTGGTCATTTAGAGGAAGTAA) and ITS2-2043R (GCTGCGTTCTTCATCGATGC). Negative controls were included for both PCR reactions. Library preparation was performed using the ALFA-SEQ DNA Library Prep Kit, and sequencing was carried out on an Illumina PE250 platform.

### Pre-processing of data

Raw sequencing data were processed using QIIME2 (V-2023.5.0, https://qiime2.org/) through the following key steps: (i) merging and demultiplexing paired-end sequences with default parameters, (ii) determining optimal trimming parameters for quality control via Figaro (https://github.com/ZymoResearch/figaro), (iii) performing denoising and error correction using the DADA2 plugin, (iv) filtering the feature table with minimum thresholds of 10 reads per feature and at least two samples per feature, (v) training a naive Bayes classifier using the fit-classifier-naive-bayes plugin against the Greengenes2 database (GG2, Version 2022.10), the UNITE database (QIIME release for Fungi 2, Version 2024.04), and the Human Oral Microbiome Database (HOMD v-15.23 16S rRNA RedSeq), (vi) taxonomic classification of sequences using the classify-sklearn algorithm against both GG2, UNITE, and HOMD databases with confidence thresholds of 0.95 for GG2 and UNITE and 0.9999 for HOMD, and (vii) retaining samples with sequencing depths of ≥14,000 reads for 16S rRNA and ≥19,500 reads for ITS, as determined by alpha-rarefaction analysis in QIIME2.

### Analysis of microbial compositions and shared species

Taxonomic composition was analyzed at the phylum and genus levels using R (version 4.4.1). Shared species were identified to assess species overlap across different groups for both bacterial and fungal communities (14). The *A*2 algorithm was applied to predict expected shared species under a null model, randomly reassigning samples to groups and permuting reads within pseudo-groups based on permutation test principles.

### Diversity and heterogeneity analysis of the esophageal microbiome

α-diversity was calculated using Hill numbers, which provide a more intuitive and statistically robust measure than conventional diversity indices. When diversity order *q* = 0, α-diversity corresponded to species richness; at *q* = 1, it was equivalent to the Shannon index; and at *q* = 2, it represented the reciprocal of the Simpson index (14–16). Wilcoxon tests were performed to assess statistical differences in α-diversity across groups.

Bray-Curtis dissimilarity was computed using Hellinger-transformed data to generate a β-diversity distance matrix, which was visualized via principal coordinate analysis (PCoA). PERMANOVA was conducted using the ADONIS function in the “vegan” package to evaluate group-wise differences in β-diversity.

To assess spatial variability and temporal stability of species distribution within communities, Type-I Taylor’s Power Law Extensions (TPLE) were applied, quantifying community spatial heterogeneity (aggregation) (14). A permutation test was used to statistically compare TPLE parameters between groups.

### Characterized microbial taxa analysis of the esophageal microbiome

Linear discriminant analysis effect size (LEfSe) was employed to identify microbiome signatures associated with different groups (17). This method integrates the nonparametric Kruskal-Wallis rank-sum test and the Wilcoxon rank-sum test to detect microbial taxa exhibiting significant intergroup variations across samples. Taxa with a linear discriminant analysis (LDA) score ≥3.0 were considered strongly associated with specific groups.

### Co-occurrence and receiver operating characteristic (ROC) analysis of the esophageal microbiome

The SparCC algorithm was applied to estimate correlations within the esophageal microbiome using 100 iterations, 1,000 bootstrap replicates, and 1,000 permutations (18). Correlations with a false discovery rate-adjusted *P* value < 0.05 and absolute correlation coefficients >0.4 were retained. Microbial co-occurrence networks at the species level were visualized in Cytoscape (version 3.10.0), with core clusters identified using the MCODE plug-in. Core nodes represented taxa interconnected by edges, while peripheral nodes were linked to core nodes but not directly to each other. The network skeleton was constructed by weighting edges based on correlation strength (19). ROC curve analysis was conducted to assess the diagnostic performance of microbiome-based markers in ESIN and ESCC.

### Predicted functional profiles of the esophageal microbiome

Functional predictions were performed using Tax4Fun2, which inferred metabolic profiles from 16S rRNA data based on KEGG Orthology (KO) annotations for prokaryotes (20). FunGuild was applied to ITS amplicon data to classify fungal taxa into functional groups within the Trophic Mode and Guild categories (21). All analyses and visualizations were conducted in R (v4.4.1).

## RESULTS

### Participant characteristics

The average ages of participants in the HC, ESIN, and ESCC groups were 64.3 ± 11.0, 63.5 ± 9.8, and 66.8 ± 5.9 years, respectively. Men constituted the majority in all groups, comprising 90.0% of the ESIN group, 91.7% of the ESCC group, and 69.2% of the HC group. Esophageal lesions predominantly occurred in the middle esophagus, accounting for 80% of cases in the ESIN group and 75% in the ESCC group. No significant differences were observed among the groups in terms of occupation (agriculturalists), educational background (below junior high school), smoking status, or alcohol flushing response (*P* > 0.05). However, significant differences were identified in alcohol consumption, preference for hot food and beverages, family history of cancer, and annual family income below 50,000 RMB (*P* < 0.05) (Table 1).

**TABLE 1:** Clinical profiles of enrolled HC, ESIN, and ESCC patients^*a*^

Characteristic	ALL (*n* = 35)	HC (*n* = 13)	ESIN (*n* = 10)	ESCC (*n* = 12)	*P* value^*b*^
Age	64.9 (9.04)	64.3 (11.0)	63.5 (9.80)	66.8 (5.94)	0.669
Gender male)	29 (82.9%)	9 (69.2%)	9 (90.0%)	11 (91.7%)	0.329
Married (yes)	31 (88.6%)	13 (100%)	8 (80.0%)	10 (83.3%)	0.293
Agriculturalist (yes)	17 (48.6%)	5 (38.5%)	3 (30.0%)	9 (75.0%)	0.09
Below junior high school (yes)	27 (77.1%)	11 (84.6%)	6 (60.0%)	10 (83.3%)	0.405
Current smoking (yes)	21 (60.0%)	5 (38.5%)	6 (60.0%)	10 (83.3%)	0.098
Alcohol flushing (yes)	21 (60.0%)	7 (53.8%)	7 (70.0%)	7 (58.3%)	0.831
Current alcohol (yes)	22 (62.9%)	4 (30.8%)	7 (70.0%)	11 (91.7%)	**0.006**
Eating vegetables daily (yes)	9 (25.7%)	1 (7.69%)	5 (50.0%)	3 (25.0%)	0.086
Eating fruit daily (yes)	3 (8.57%)	0 (0.00%)	2 (20.0%)	1 (8.33%)	0.178
Eating fast (yes)	12 (34.3%)	3 (23.1%)	6 (60.0%)	3 (25.0%)	0.147
Eating a salty diet (yes)	8 (22.9%)	1 (7.69%)	5 (50.0%)	2 (16.7%)	0.068
Family history of cancer (yes)	3 (8.57%)	0 (0.00%)	3 (30.0%)	0 (0.00%)	**0.018**
Preference for hot food and beverages (yes)	8 (22.9%)	0 (0.00%)	5 (50.0%)	3 (25.0%)	**0.009**
Annual family income below 50,000 RMB (yes)	22 (62.9%)	5 (38.5%)	6 (60.0%)	11 (91.7%)	**0.020**

^*a*^ Fisher’s exact test for categorical variables and ANOVA for continuous variables. ESCC, esophageal squamous cell carcinoma; ESIN, esophageal squamous intraepithelial neoplasia; HC, healthy controls.

^*b*^ The bold values indicate statistical significance where the *P* value < 0.05.

### Analysis of microbial compositions and shared species

**Fig 2:**
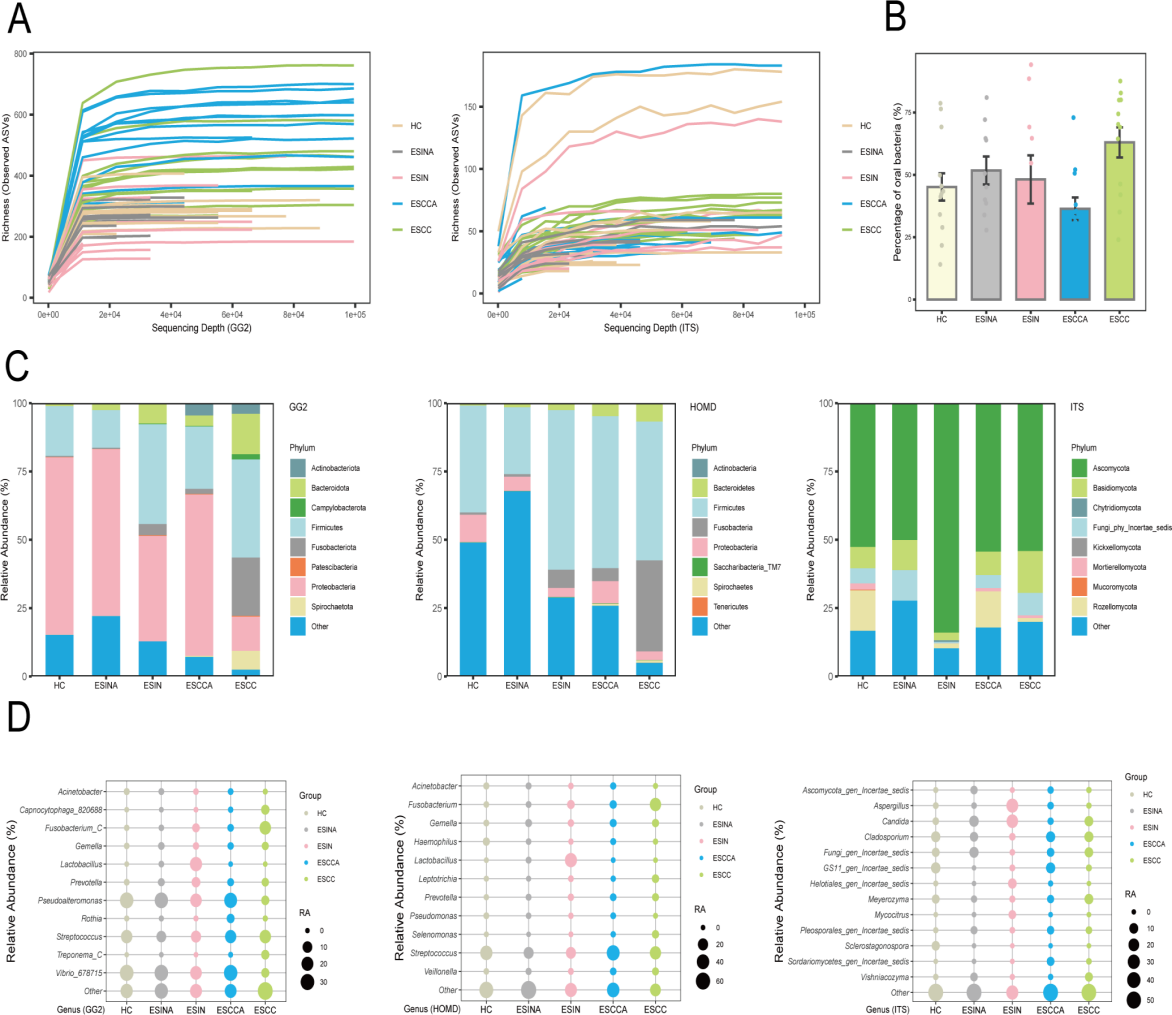
Rarefaction curves of 16S rRNA and ITS (**A**). Percentage distribution of oral microbiome across five groups (HC, ESINA, ESIN, ESCCA, and ESCC) (**B**). Relative abundance of bacteria, oral microbime, and fungi at the phylum (**C**) and genus levels (**D**) across the five groups. GG2, the Greengenes2 Database; HOMD, the Human Oral Microbiome Database; ITS, internal transcribed spacer; ESCC, esophageal squamous cell carcinoma; ESCCA, esophageal squamous cell carcinoma adjacent tissues; ESIN, esophageal squamous intraepithelial neoplasia; ESINA, esophageal squamous intraepithelial neoplasia adjacent tissues; HC, healthy controls.

Rarefaction curve analysis confirmed sufficient sequencing depth for most samples in both 16S rRNA and ITS data sets (Fig. 2A). Given the established relevance of the oral microbiome in ESCC, the proportion of oral microbiome (bacteria annotated as oral microbiome in HOMD) was examined across groups (22). A progressive increase in oral microbiome prevalence was observed with disease severity (HC < ESIN < ESCC). The ESCC group exhibited a higher proportion of oral microbiome compared to ESCCA, whereas the ESIN and ESINA groups displayed the opposite trend (Fig. 2B).

At the phylum level, bacterial communities in the HC and ESIN groups were primarily composed of *Proteobacteria* and *Firmicutes*, albeit with differing relative abundances. In contrast, the ESCC group was dominated by *Firmicutes* and *Fusobacteriota*. Similarly, the ESINA and ESCCA groups exhibited a bacterial composition resembling that of HC and ESIN, with *Proteobacteria* and *Firmicutes* as the predominant phyla (Fig. 2C, GG2). The composition of the oral microbiome followed a similar trend, albeit with variations in relative proportions (Fig. 2C, HOMD). Regarding fungal communities, *Ascomycota* was the dominant phylum across all groups, with ESIN exhibiting the highest relative abundance (83.98%) (Fig. 2C, ITS).

Microbial composition at the genus level is visualized in Fig. 2D. In the HC group, the three most abundant bacterial genera were *Vibrio* (30.3%), *Pseudoalteromonas* (29.2%), and *Streptococcus* (17.1%). The ESIN group was characterized by an enrichment of *Lactobacillus* (21.0%), Vibrio (18.1%), and *Pseudoalteromonas* (17.7%). In ESCC, *Fusobacterium* (17.7%), *Streptococcus* (17.4%), and *Capnocytophaga* (5.5%) were predominant. The ESINA and ESCCA groups exhibited a bacterial composition largely similar to HC, dominated by *Vibrio*, *Pseudoalteromonas*, and *Streptococcus*, albeit with differences in relative abundance. Notably, *Lactobacillus*, *Fusobacterium*, *Streptococcus*, and *Capnocytophaga* are recognized as members of the oral microbiome. Interestingly, while fungal communities in HC, ESCC, ESINA, and ESCCA exhibited similar profiles, the ESIN group demonstrated a distinct composition, with a notable enrichment of *Aspergillus* (27.4%) and *Candida* (24.7%).

A total of 2,314, 2,004, and 573 amplicon sequence variants (ASVs) were obtained from the GG2, HOMD, and UNIT databases, respectively, after sequence denoising across all samples. Fig. S1 illustrates the shared species analysis results for the various comparison groups in both bacteria and fungi. The ESIN and ESINA groups exhibited the fewest shared species, comprising 711 bacterial and 106 fungal ASVs (Table S1). In contrast, the ESCC and ESCCA groups displayed a higher number of shared species, particularly 1,545 bacterial ASVs. Additionally, in terms of bacterial ASVs, the ESCC and ESIN groups shared more ASVs compared to the ESIN and HC groups (1,093 vs. 828), a pattern not observed in fungi.

### Analyzing diversity and heterogeneity of the esophageal microbiome

**Fig 3:**
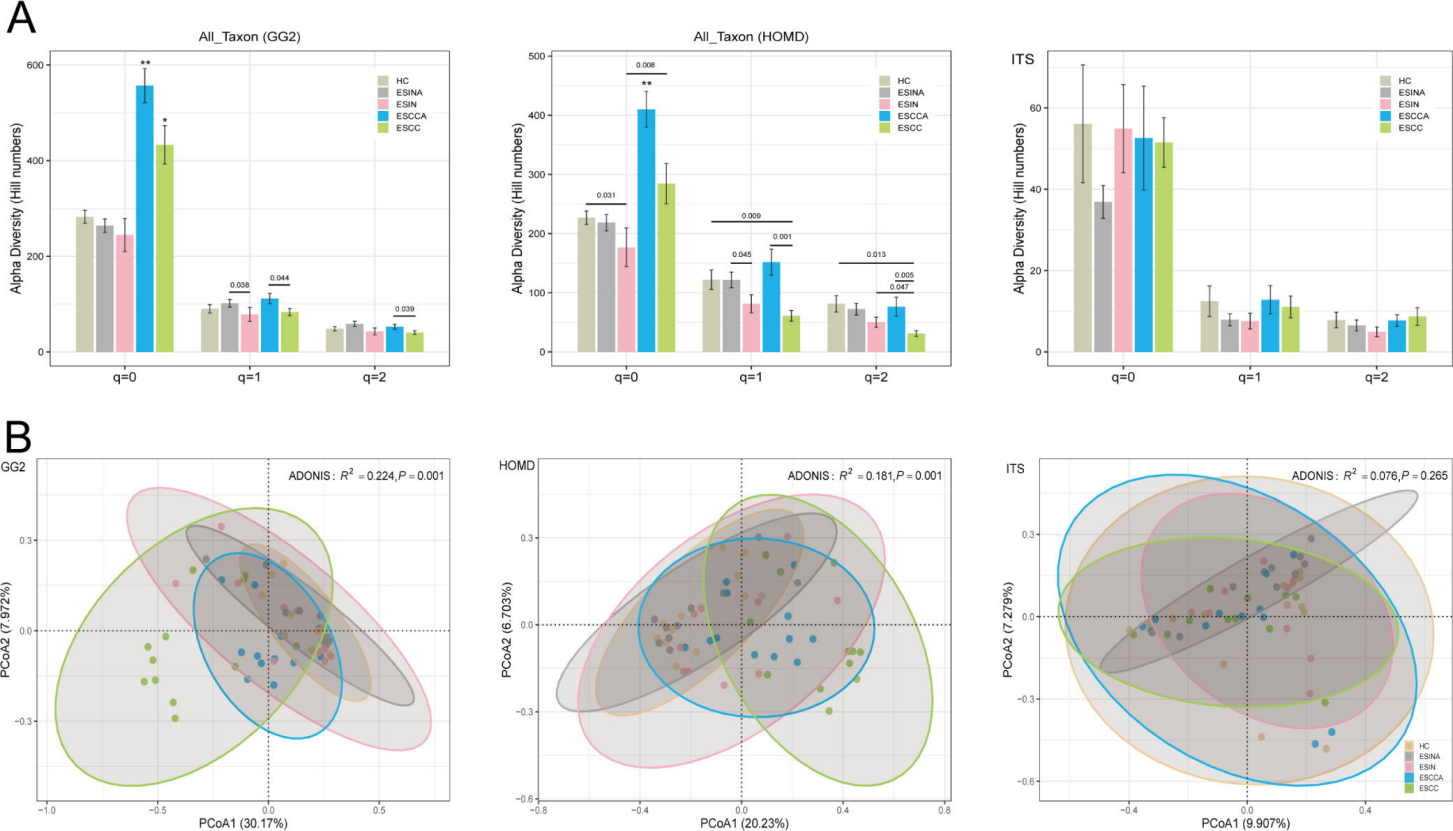
(**A**) Mean Hill numbers (α-diversity) for bacteria, oral microbiome, and fungi across five groups (HC, ESINA, ESIN, ESCCA, and ESCC). “–” denotes statistical differences between two groups (*P* < 0.05). “*” and “**” indicate significant differences compared to all other groups. (**B**) PCoA plots showing β-diversity based on Bray-Curtis distance for bacteria (*P* = 0.001), oral microbiome (*P* = 0.001), and fungi (*P* = 0.265) across the five groups. GG2, the Greengenes2 Database; HOMD, the Human Oral Microbiome Database; ITS, internal transcribed spacer; ESCC, esophageal squamous cell carcinoma; ESCCA, esophageal squamous cell carcinoma adjacent tissues; ESIN, esophageal squamous intraepithelial neoplasia; ESINA, esophageal squamous intraepithelial neoplasia adjacent tissues; HC, healthy controls.

The α-diversity of the esophageal microbiome was assessed using Hill numbers for both bacteria and fungi. Table S2-1 presents the *mean* and *standard error* of α-diversity for each group at diversity orders *q* = 0–2, along with statistical tests for eight distinct groups listed in Table S2-2. Notably, individuals with ESCC exhibited significantly higher bacterial richness (*q* = 0), regardless of subgroup classification (ESCC or ESCCA), compared to other groups. Moreover, the mean Hill number was higher in the HC group compared to the ESIN and ESCC groups at *q* = 1 and *q* = 2. Similarly, bacterial diversity (*q* = 1 and *q* = 2) was greater in adjacent lesion/cancer tissue compared to lesion/cancer tissue (ESINA > ESIN, ESCCA > ESCC). However, except for *q* = 2, no statistically significant differences were observed between the ESINA and ESIN groups, while other comparisons showed statistical significance (Fig. 3A GG2). Furthermore, the α-diversity trend of the oral microbiome mirrored that observed in bacterial diversity (Fig. 3A, HOMD). In contrast to bacteria, the HC group exhibited the greatest fungal richness and diversity (*q* = 0–2), with lower diversity observed in the ESIN and ESCC groups. Except for *q* = 2, fungal diversity in ESCC was marginally higher than in HC, though no statistical differences were found (Fig. 3A, ITS).

Figure 3B visualizes the β-diversity of bacterial and fungal communities across clinical groups (HC, ESINA, ESIN, ESCCA, and ESCC) via PCoA based on Hellinger-transformed Bray-Curtis dissimilarity matrices. Bacterial communities across the five groups formed distinct clusters (*R*^2^ = 0.224, *P* = 0.001). Similarly, oral microbiome communities showed distinct separation among the five groups (*R*^2^ = 0.181, *P* = 0.001). Notably, no significant difference in bacterial β-diversity was observed between the HC and ESIN groups, while a notable difference was observed between the two groups for the oral microbiome (Table 2). In contrast, fungal β-diversity did not show significant separation (*R*^2^ = 0.076, *P* = 0.228).

**TABLE 2:** Differences in β-diversity across the groups with ANOSIM test

	Comparison	*R* ^2^	*P* value	*P* adjusted^*b*^
GG2	HC vs ESINA	0.045	0.419	0.424
	HC vs ESIN	0.070	0.057	0.071
	HC vs ESCCA	0.111	0.001	**0.003**
	HC vs ESCC	0.285	0.001	**0.003**
	ESINA vs ESIN	0.050	0.424	0.424
	ESINA vs ESCCA	0.107	0.005	**0.008**
	ESIN vs ESCC	0.173	0.005	**0.008**
	ESCCA vs ESCC	0.155	0.001	**0.003**
HOMD	HC vs ESINA	0.049	0.244	0.244
	HC vs ESIN	0.074	0.015	**0.019**
	HC vs ESCCA	0.101	0.001	**0.003**
	HC vs ESCC	0.214	0.001	**0.003**
	ESINA vs ESIN	0.067	0.116	0.129
	ESINA vs ESCCA	0.115	0.002	**0.003**
	ESIN vs ESCC	0.137	0.002	**0.003**
	ESCCA vs ESCC	0.105	0.002	**0.003**
ITS	HC vs ESINA	0.058	0.102	0.370
	HC vs ESIN	0.053	0.176	0.370
	HC vs ESCCA	0.039	0.522	0.580
	HC vs ESCC	0.043	0.359	0.580
	ESINA vs ESIN	0.053	0.457	0.580
	ESINA vs ESCCA	0.056	0.173	0.370
	ESIN vs ESCC	0.047	0.487	0.580
	ESCCA vs ESCC	0.031	0.976	0.976

^*a*^ ESCC, esophageal squamous cell carcinoma; ESCCA, esophageal squamous cell carcinoma adjacent tissues; ESIN, esophageal squamous intraepithelial neoplasia; ESINA, esophageal squamous intraepithelial neoplasia adjacent tissues; GG2, the Greengenes2 Database; HC, healthy controls; HOMD, the Human Oral Microbiome Database; ITS, internal transcribed spacer.

^*b*^ The bold values indicate statistical significance where the *P* value < 0.05.

**Fig 4:**
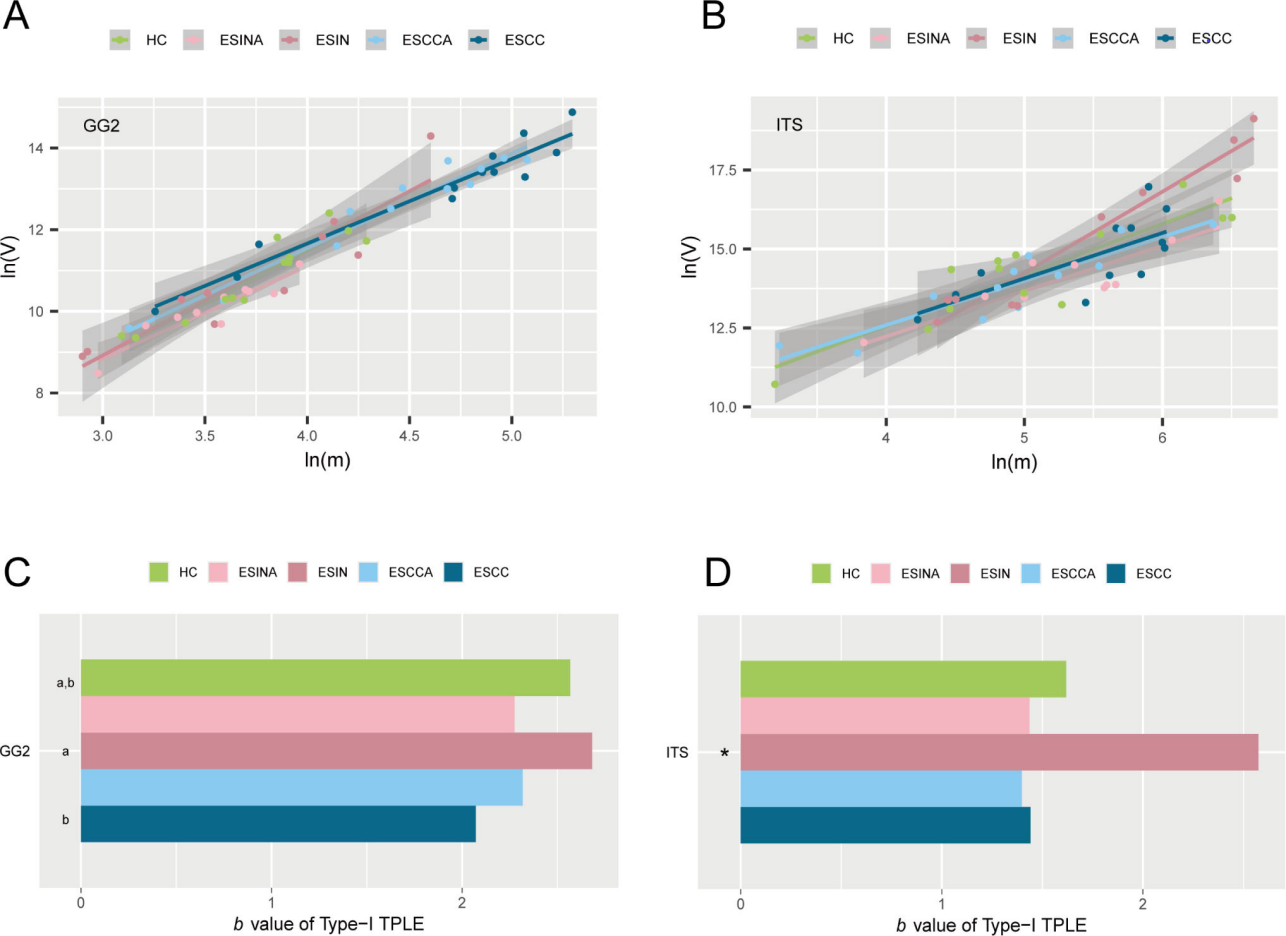
Type-I TPLE was applied to assess community spatial heterogeneity for bacteria (**A**) and fungi (**B**) across five groups (HC, ESINA, ESIN, ESCCA, and ESCC). (**C**) *b* Values for bacteria and (**D**) *b* Values for fungi. Groups sharing the same letter show no statistically significant difference in *b* values, while “*” indicates significant differences compared to all other groups. GG2, the Greengenes2 Database; HOMD, the Human Oral Microbiome Database; ITS, internal transcribed spacer; ESCC, esophageal squamous cell carcinoma; ESCCA, esophageal squamous cell carcinoma adjacent tissues; ESIN, esophageal squamous intraepithelial neoplasia; ESINA, esophageal squamous intraepithelial neoplasia adjacent tissues; HC, healthy controls; TPLE, Taylor’s Power Law Extensions.

Given the alignment between oral microbiome patterns and overall bacterial community profiles, as demonstrated in previous analyses, separate analyses for the oral microbiome were not conducted in subsequent steps. The spatial heterogeneity across groups was assessed using Type-I TPLE for both bacteria and fungi. The Type-I TPLE model exhibited strong fit for esophageal bacteria and fungi (*P* < 0.05), as shown in Fig. 4A and B. Table S3-1 lists the Type-I TPLE parameters for esophageal bacteria and fungi across the five groups, including *b* values, ln(*a*), correlation coefficients (*R*), *P* values, and sample sizes (*n*). Higher *b* values indicate greater spatial heterogeneity among groups. Significant differences in *b* values were observed for bacteria and fungi. For example, in the ESIN group, the *b* values for bacteria and fungi were 2.682 and 2.574, respectively. Permutation tests were used to detect differences in TPLE parameters across all groups (Table S3-2). The ESIN group exhibited significantly higher *b* values for bacteria compared to the ESCC group. For fungi, the ESIN group demonstrated the highest *b* values among all groups (Fig. 4C and D).

### Characterized microbial taxa analysis of the esophageal microbiome

The microbiome enrichment in each comparison group was analyzed using LEfSe. In bacterial comparisons between the HC, ESIN, and ESCC groups, *Vibrio*, *Pseudoalteromonas*, and *Acinetobacter* were the most prominent genera in the HC group. *Lactobacillus* was more abundant in the ESIN group, while *Fusobacterium*, *Streptococcus*, *Treponema*, *Gemella*, *Veillonella*, and *Capnocytophaga* were more prevalent in the ESCC group (Fig. S2A). Distinct dominant bacteria were observed between the ESCC and ESCCA groups, as well as between the ESIN and ESINA groups.

Regarding fungal composition, *Aspergillus* was the dominant fungus in the ESIN group, while *Alternaria*, *Neosetophoma*, and *Monascus* were more prevalent in the ESCC group. *Coniochaeta* and *Sclerostagonospora* were more abundant in the HC group (Fig. S2B). Notably, the prominence of *Aspergillus* in the ESIN group was also evident in the comparison with the ESINA group, while no key fungal species were identified in the ESCC versus ESCCA comparison.

### Co-occurrence and ROC analysis of esophageal microbiome

**Fig 5:**
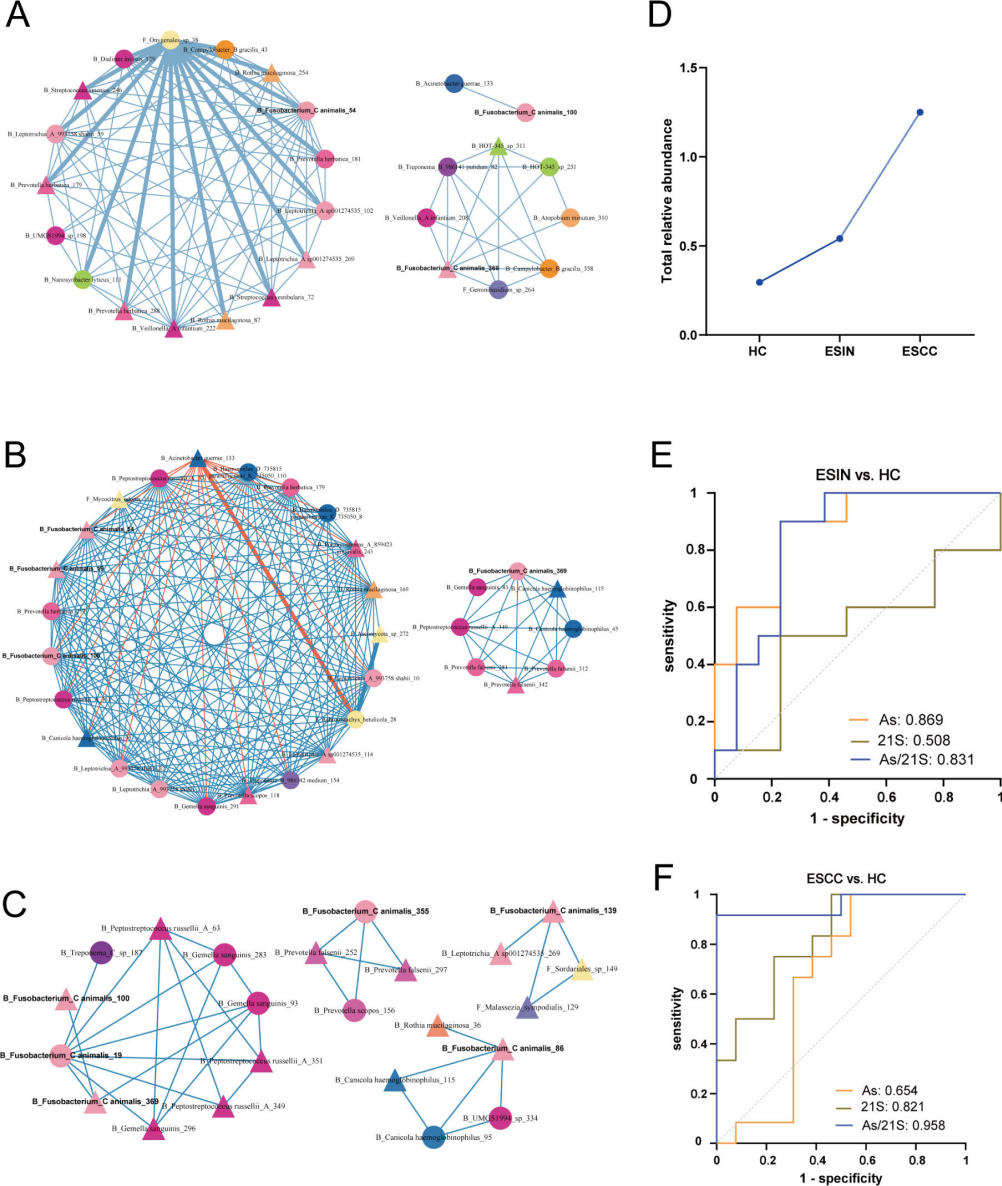
Co-occurrence network involving interactions with *Fusobacterium animalis* in the HC (**A**), ESIN (**B**), and ESCC (**C**) groups. Core nodes are represented by circles, peripheral nodes by triangles, and each node represents a distinct species. Bold edges indicate the network backbone, with orange edges denoting negative correlations and blue edges indicating positive correlations. Different colors represent microbial phyla. (**D**) Shows the total relative abundance of the 21 species across the HC, ESIN, and ESCC groups. ROC curves assess the ability to distinguish ESIN (**E**) and ESCC (**F**) patients from HC for *Aspergillus* (As), 21 species (21S), and the combined *Aspergillus*/21 species (As/21S). 21S, 21 species; AS, Aspergillus; ESCC, esophageal squamous cell carcinoma; ESIN, esophageal squamous intraepithelial neoplasia; HC, healthy controls.

To explore interactions among esophageal bacterial and fungal species, microbial co-occurrence networks for the HC, ESIN, and ESCC groups were visualized (Fig. S3). The ESCC group exhibited the sparsest relationships among microbiomes, characterized by fewer microbial species, fewer edges, and lower average connectivity between network nodes. Species interactions varied across networks. In the HC group, *Fusobacterium animalis* (*F. animal*) positively correlated with *Acinetobacter guerra (A.guerra)* (Fig. 5A), but this correlation was negative in the ESIN group (Fig. 5B). No correlation was observed between *F. animalis* and *A. guerra* in the ESCC group (Fig. 5C). *Candida albicans* (*C. albicans*), frequently observed in patients with premalignant lesions or early-stage esophageal cancer (23), showed a negative correlation with *Ralstonia mannitolilytica* and 28 fungal ASVs in the HC group. In the ESIN group, it demonstrated a positive correlation only with an unannotated fungal ASV. In the ESCC group, *C. albicans* exhibited a positive correlation with *Rothia mucilaginosa*.

Considering disease progression and the relatively sparse ESCC network, species with common interactions in the ESIN and HC networks were identified and confirmed to be present in the ESCC network. A total of 21 species were identified, including 18 bacterial and 3 fungal species (Fig. S4). Several pathogenic species, such as *Porphyromonas gingivalis*, *Fusobacterium animalis*, *Haemophilus parainfluenzae*, *Rothia mucilaginosa*, and *Mogibacterium pumilum*, were among them. With disease progression, the overall relative abundance of these 21 species increased (Fig. 5D).

Using *Aspergillus*, the most enriched species in the ESIN group as identified by LEfSe analysis, in combination with 21 species, ROC curves were generated to determine cutoff values for distinguishing the ESCC/ESIN groups from the HC group (Fig. 5E and F). The area under the ROC curve (AUC) for diagnosing ESIN versus HC was 0.869 for *Aspergillus*, 0.508 for the 21 species, and 0.831 for the combination of *Aspergillus*/21 species. For differentiating ESCC from HC, the AUC values were 0.654, 0.821, and 0.958, respectively. *Aspergillus* showed strong diagnostic potential for the ESIN group, while the combination of *Aspergillus* and 21 species significantly enhanced diagnostic accuracy for the ESCC group. These 21 species were thus identified as a potential disease-related functional group.

### Predicted functional profiles of the esophageal microbiome

**Fig 6:**
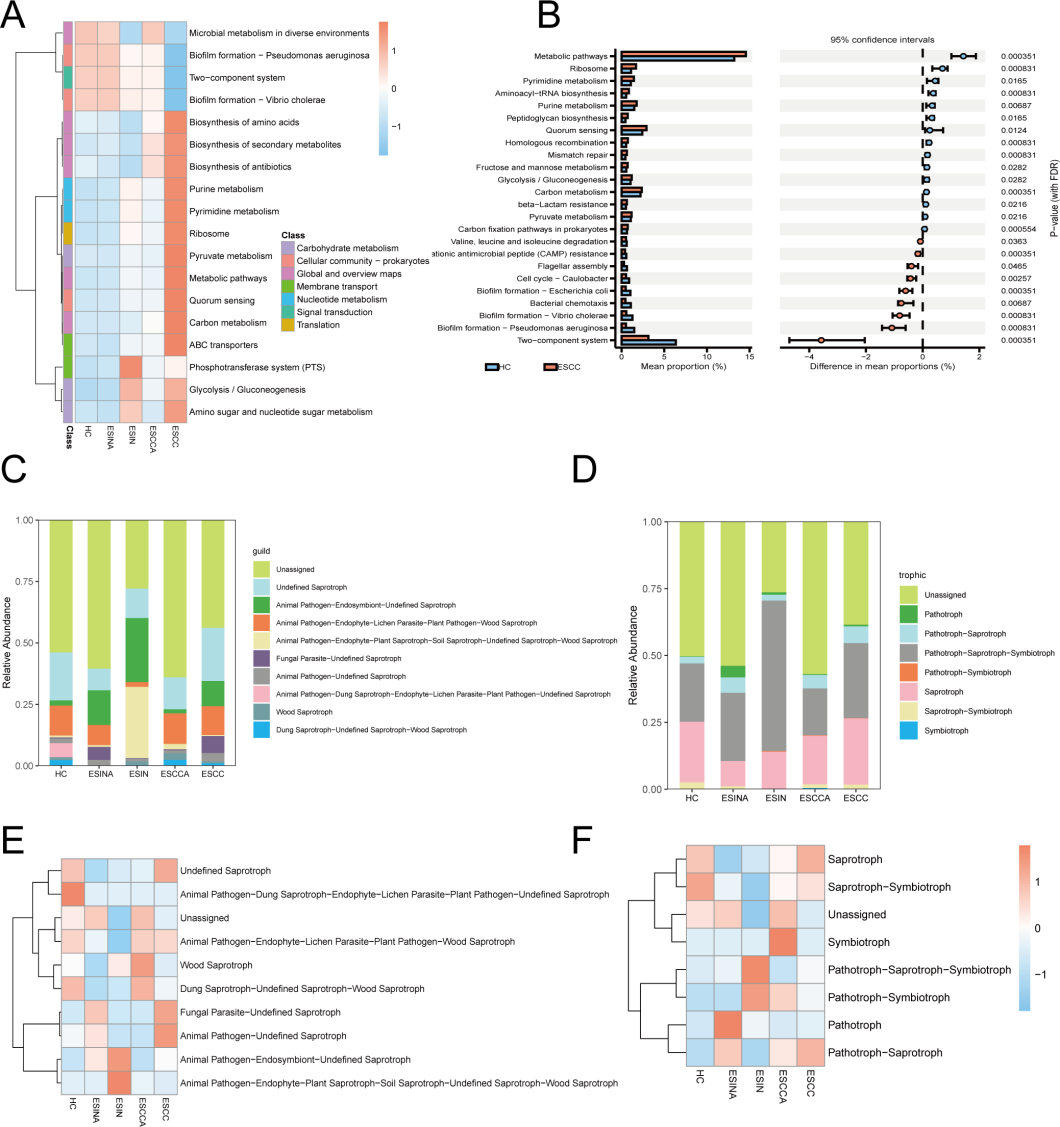
(**A**) Heatmap of predicted bacterial functional expression across five groups (HC, ESINA, ESIN, ESCCA, and ESCC). (**B**) Differential pathway analysis between HC and ESCC. (C–F) Fungal functional predictions using FUNGuild: (**C**) guild classification, (**D**) trophic mode classification, (**E, F**) heatmaps of predicted functional expression under fungal guilds (**E**), and trophic modes (**F**) across five groups. ESCC, esophageal squamous cell carcinoma; ESCCA, adjacent tissues of esophageal squamous cell carcinoma; ESIN, esophageal squamous intraepithelial neoplasia; ESINA, adjacent tissues of esophageal squamous intraepithelial neoplasia; HC, healthy controls.

Tax4Fun2-based bacterial functional predictions revealed predominant enrichment of tumor-associated pathways in the ESCC group (Fig. 6A). Corrective differential abundance analysis identified variations in 24 pathways between the HC and ESCC groups (Fig. 6B). FUNGuild fungal functional annotation classified communities into 48 ecological guilds across eight trophic modes, with unclassified taxa dominating (Fig. 6C and D). Comparative heatmaps visualized functional predictions using dual fungal annotation strategies: ecological guilds and trophic modes (Fig. 6E and F).

## DISCUSSION

This study employed high-throughput sequencing of 16S rRNA and ITS to investigate bacterial and fungal composition, diversity, similarity, heterogeneity, interactions, and potential functions throughout the progression of ESCC. Our results highlighted that ESCC progression was associated with dysbiosis in the esophageal resident microbiome, affecting both bacterial and fungal populations. Notably, individuals with precancerous lesions (ESIN) exhibited significant heterogeneity. The interactions between bacteria and fungi, along with the enriched functional pathways during ESCC progression, were distinct.

Alcohol consumption, preference for hot drinks and foods, family history of cancer, and low household income have been established as risk factors for ESCC (24). Among these, alcohol consumption and the intake of hot drinks and foods may influence the gut microbiome. Mutlu et al. reported that alcoholics exhibit gut microbiota dysbiosis, characterized by lower *Bacteroidetes* and higher *Proteobacteria* abundances (25). Similarly, studies on heat stress effects on the human gut microbiota are limited, mainly based on animal models, which show that acute heat stress increases the *Firmicutes*-to-*Bacteroidetes* ratio, with elevated *Firmicutes* and reduced *Bacteroidetes* (26). However, in the present study, no such microbiome alterations were observed. Therefore, it remains unclear whether these factors influence the microbiome associated with ESCC progression, and further experiments are necessary to verify this hypothesis.

Previous research demonstrated the significant role of the oral microbiome in esophageal cancer onset and progression (22), which was corroborated by our findings. A gradual increase in the proportion of the oral microbiome was observed as the disease advanced. Additionally, individuals with ESCC (including both the ESCC and ESCCA groups) exhibited the highest bacterial richness, while the HC group showed the highest fungal richness. At *q* = 1, the HC group exhibited the highest bacterial and fungal α-diversity. However, at *q* = 2, the HC group retained the highest bacterial α-diversity, whereas the ESCC group exhibited the highest fungal α-diversity. These findings suggest that ESCC progression influences the diversity of the esophageal microbiome, aligning with reports by Jiang et al. and Rao et al., who noted significantly reduced bacterial and fungal α-diversity in ESCC compared to control groups (10, 27). Furthermore, a notable bacterial β-diversity difference was observed between the ESCC group and other groups, consistent with previous studies, while no significant variations were noted in fungal communities (10). These results indicate that ESCC progression may exert a more pronounced impact on bacterial communities than on fungal ones.

In the analysis of oral microbiome β-diversity, significant differences were observed between the HC and ESIN groups. However, no significant separation in the β-diversity patterns of bacteria was noted between these two groups. Additionally, in the analysis of shared species between bacteria and fungi, the ESIN group demonstrated the lowest degree of species sharing when compared to the ESINA group. Within the TPLE model, both fungi and bacteria in the ESIN group exhibited significantly higher *b* values than in the other groups, suggesting a notable degree of heterogeneity. These findings indicate that the ESIN group displayed a high level of variability. Furthermore, the oral microbiome could serve as a distinguishing factor between health and early-stage esophageal pathology, although further validation is required to establish its potential as a biomarker for early ESCC detection.

The ESIN group showed a high abundance of certain bacteria, including *Lactobacillus*, *Vibrio*, and *Pseudoalteromonas*. However, only *Lactobacillus* was identified as a representative taxon of the ESIN group through LEfSe analysis. While *Lactobacillus* is commonly regarded as a beneficial microorganism in the human gastrointestinal tract, it has also been found to be more abundant in cancers such as gastric cancer, breast cancer, and head and neck squamous cell carcinoma (28–31). Sonveaux et al. suggested that *Lactobacillus* may produce metabolites that serve as an energy source for tumor growth and angiogenesis (32). Additionally, *Aspergillus* emerged as the most significantly enriched fungal genus in the ESIN group. This oral commensal has previously been linked to immunosuppression in lung adenocarcinoma through the β-glucan/Dectin-1/CARD9/IL-1β axis (33, 34). While our ITS sequencing data cannot establish causality in esophageal carcinogenesis, the observed enrichment of *Aspergillus* raises an intriguing hypothesis: *Aspergillus*-derived β-glucan may interact with Dectin-1 receptors on esophageal mucosal immune cells, potentially creating a microenvironment conducive to early neoplastic progression. This hypothesis warrants further validation in functional models.

In the ESCC group, *Fusobacterium*, *Streptococcus*, and *Capnocytophaga* were identified as the dominant bacterial genera, commonly found in the oral microbiome and previously reported to be enriched in ESCC (10, 35–37). *Fusobacterium nucleatum* (*F. nucleatum*) is well known for promoting ESCC proliferation by enhancing the expression of interleukin-32/proteinase 3, which activates the PI3K/AKT signaling pathway in both *in vivo* and *in vitro* models (35). However, our study identified a subspecies of *F. nucleatum*, *F. animalis* (*F. nucleatum animalis*, Fna), an emerging oral pathogen prevalent in odontogenic abscesses and colorectal cancer (CRC) (38, 39). A recent study revealed that Fna adhered to and invaded CRC tumor tissues through its Fap2 protein, with the expansion, recombination, and horizontal transfer of its Fic gene family enhancing its pathogenicity. Furthermore, Fna upregulated its pathogenic-related genes during interactions with host cells, contributing to CRC progression (40). Notably, a large-scale study of *F. nucleatum* strains from CRC and non-cancer oral samples found that Fna splits into two clades: Fna C1, confined to the oral cavity, and Fna C2, which dominates the CRC tumor niche (41). Given its oncogenic potential, Fna C2 warrants prioritized investigation into its clade-specific contributions to ESCC pathogenesis. Additionally, *Alternaria*, *Neosetophoma*, and *Monascus* were identified as key fungi in the ESCC group. However, few studies have explored the role of *Alternaria* in human diseases, with one showing that exposure to *Alternaria* increases the risk of allergic conditions. Therefore, further investigation into the roles of these bacteria and fungi in ESCC development is needed (42).

Co-occurrence analysis revealed a reduction in microbial interactions as disease severity increased, consistent with previous findings in tongue squamous cell carcinoma (43). Co-occurrence network analysis identified a potential disease-related functional group consisting of multiple pathogenic species. As their relative abundance increased with disease progression, ROC analysis demonstrated their diagnostic value in ESIN and ESCC. These potential disease functional groups likely maintain dynamic stability in the esophagus through various interactions, including nutrient exchange, resource competition, antimicrobial production, iron competition, quorum sensing, inhibition of fungal growth by bacterial short-chain fatty acids, and cooperative nutrient sharing (44, 45). Alterations in the interactions among these species may disrupt the esophageal microbial community, ultimately contributing to the progression of ESCC.

Functional analysis of bacterial communities revealed upregulated cancer-related pathways and significantly downregulated biofilm formation pathways in the ESCC group, suggesting a potential link between biofilm loss and cancer progression. Fungal community analysis highlighted shifts in ecological guilds and trophic types across esophageal tissues, reflecting changes in the esophageal environment and host-microbiome interactions.

This study provides a comprehensive analysis of bacterial and fungal characteristics, interkingdom interactions, and enriched functional pathways during ESCC progression. Key findings, such as the relative abundance of dominant microbiomes, microbial diversity, similarity, and heterogeneity across different groups, may serve as potential reference values for characterizing various stages of ESCC progression. However, certain limitations should be acknowledged. Firstly, all participants were from a single region, limiting the ability to perform stratified analyses of epidemiological factors. Although participants with five different esophageal statuses were included, specific differentiation within ESIN levels was not performed, given the practical requirements for early detection. Additionally, estimating statistical power and effect size remains a challenge in microbiome research (46). Due to difficulties in early ESCC diagnosis and limited access to ESIN samples, the sample size for this exploratory study was based on comparable studies. To achieve more precise results, future research should adopt a larger sample size, a multicenter approach, and sufficient power to assess the utility of participants across different lesion types. Furthermore, to better understand the mechanisms linking microbiomes to the host, future studies should include both *in vivo* and *in vitro* experiments.

In conclusion, this study uncovered stage-specific characteristics of the esophageal microbiome in ESCC progression. ESIN exhibited higher heterogeneity with *Lactobacillus*/*Aspergillus* enrichment, contrasting with *Fusobacterium*/*Alternaria* dominance in ESCC. Early divergence in oral microbiome β-diversity preceded esophageal bacterial alterations. Additionally, the co-occurrence network was sparser at ESCC stages, with the *Aspergillus*-to-pathogenic functional group ratio enhancing ESCC diagnostic accuracy. ESCC-associated bacterial pathways (carbohydrate and nucleotide metabolism) and fungal functional shifts implicated microenvironment reprogramming. These microbial signatures offer actionable biomarkers for early detection and provide mechanistic insights into ESCC pathogenesis.

## Data Availability

All raw sequencing reads are available in the NCBI BioProject database (PRJNA1229471) (https://www.ncbi.nlm.nih.gov/sra/PRJNA1229471).

## SUPPLEMENTAL MATERIAL

Supplemental material spectrum.03137-24-s0001.pdf Tables S1 to S3; Fig. S1 to S4.

Supplemental data spectrum.03137-24-s0002.pdf Sampling workflow and quality control measures.

ASM does not own the copyrights to Supplemental Material that may be linked to, or accessed through, an article. The authors have granted ASM a non-exclusive, world-wide license to publish the Supplemental Material files. Please contact the corresponding author directly for reuse.
