# Supplementary material for: Esophageal cancer and precancerous lesions: focus on resident bacteria and fungi
Source: Microbiol Spectr. 2025 May 20;13(7):e03137-24. doi: 10.1128/spectrum.03137-24 (PMC12210859; doi:10.1128/spectrum.03137-24)
Supplement: Supplemental material — Tables S1 to S3; Fig. S1 to S4. [file spectrum.03137-24-s0001.pdf]

Online Supplementary Information (OSI) for: Esophageal Cancer and Precancerous Lesions: Focus on Resident Bacteria and Fungi

List of Online Supplementary Tables (OST)

Table S1. The results from the analysis of shared species between different groups

Table S2-1. The *Mean* and S*tandard Error* of the Hill numbers (α-diversity) for different groups

Table S2-2. The *P* values of Wilcoxon test in Hill numbers (α-diversity) for different groups

Table S3-1. The Type–I TPLE parameters for the community spatial heterogeneity of different groups

Table S3-2. The results of the permutation tests for Type-I TPLE parameters for all pairwise comparisons

List of Online Supplementary Figures (OSF)

Fig. S1 The bar graphs show observed and expected shared speices (*A2* algorithm) for bacterial (A) and fungal (B) comparisons across HC, ESINA, ESIN, ESCCA, and ESCC groups. The "☆" represent a statistical difference between the two cohorts in expected shared speices. HC: Health controls. ESINA: Esophageal squamous intraepithelial neoplasia adjacent tissues. ESIN: Esophageal squamous intraepithelial neoplasia. ESCCA: Esophageal squamous cell carcinoma adjacent tissues. ESCC: Esophageal squamous cell carcinoma. ASVs: Amplicon sequence variants.

Fig. S2 LEfSe analysis showed the most abundant taxa of bacteria (A) and fungi (B) in the genus level among HC, ESIN, and ESCC groups. LEfSe: linear discriminant analysis effect size. HC: Health controls. ESIN: Esophageal squamous intraepithelial neoplasia. ESCC: Esophageal squamous cell carcinoma.

Fig. S3 The co-occurrence analysis between esophageal bacteria and fungi in HC (A), ESIN (B), and ESCC (C) groups. Circles represent core nodes. triangles indicate peripheral nodes. Each node represents a distinct species. bold edges form the backbone. The orange edges show negative correlations. blue edges show positive correlations and different colors represent microbial phyla. HC: Health controls. ESIN: Esophageal squamous intraepithelial neoplasia. ESCC: Esophageal squamous cell carcinoma.

Fig. S4 The shared a bacterial-fungal co-occurrence network in HC (A), ESIN (B), and ESCC (C) groups. Circles represent core nodes. triangles indicate peripheral nodes. Each node represents a

distinct species. bold edges form the backbone. The orange edges show negative correlations. blue edges show positive correlations,and different colors represent microbial phyla. HC: Health controls. ESIN: Esophageal squamous intraepithelial neoplasia. ESCC: Esophageal squamous cell carcinoma.

Table S1. The results from the analysis of shared species between different groups[a]

	Comparsions	Observed Shared ASVs	Reads randomization		Samples randomization	
			Expected Shared ASVs	*P* value[b]	Expected Shared ASVs	*P* value[b]
**GG2**	HC vs. ESINA	847	1660.091	**0.000**	844.116	0.531
	HC vs. ESIN	776	1688.136	**0.000**	828.373	**0.032**
	HC vs. ESCCA	1150	2206.046	**0.000**	1427.814	**0.000**
	HC vs. ESCC	858	2056.861	**0.000**	1257.952	**0.000**
	ESINA vs. ESIN	711	1598.536	**0.000**	710.757	0.473
	ESIN vs. ESCC	814	1884.517	**0.000**	1092.949	**0.000**
	ESCCA vs. ESCC	1423	2188.986	**0.000**	1544.908	**0.010**
	Difference rate			(7/7) 100.0%		(5/7) 71.4%
**ITS**	HC vs. ESINA	92	423.834	**0.000**	158.901	**0.028**
	HC vs. ESIN	213	475.236	**0.000**	210.283	0.249
	HC vs. ESCCA	261	461.682	**0.000**	239.035	0.436
	HC vs. ESCC	141	492.145	**0.000**	203.664	0.088
	ESINA vs. ESIN	110	372.769	**0.000**	105.523	0.776
	ESIN vs. ESCC	177	426.570	**0.000**	157.448	0.870
	ESCCA vs. ESCC	151	480.785	**0.000**	155.718	0.359
	Difference rate			(7/7) 100.0%		(1/7) 14.3%

[a]GG2: Greengenes2 database. ITS: Internal Transcribed Spacer. ASVs: amplicon sequence variants. HC: Healthy Controls. ESIN: Esophageal squamous intraepithelial neoplasia. ESCC: Esophageal squamous cell carcinoma. ESINA: Esophageal squamous intraepithelial neoplasia adjacent tissues. ESCCA: Esophageal squamous cell carcinoma adjacent tissues.

[b]The bold values indicate statistical significance where the P value < 0.05.

Table S2-1. The *mean* and *standard error* of the Hill numbers (α-diversity) for different groups[a]

	Group	Statistics	*q* = 0	*q* = 1	*q* = 2
**GG2**	HC	*Mean*	282.615	90.150	48.338
		Std. Err.	13.583	8.580	4.477
	ESINA	*Mean*	264.100	101.910	58.685
		Std. Err.	14.099	8.106	5.277
	ESIN	*Mean*	244.600	78.398	42.890
		Std. Err.	34.408	14.646	7.048
	ESCCA	*Mean*	556.667	111.736	52.577
		Std. Err.	35.671	10.754	5.227
	ESCC	*Mean*	433.000	83.351	40.282
		Std. Err.	40.060	7.481	3.862
**HOMD**	HC	*Mean*	226.769	122.061	81.311
		Std. Err.	11.329	16.536	13.893
	ESINA	*Mean*	218.500	121.746	72.418
		Std. Err.	14.056	13.214	9.823
	ESIN	*Mean*	176.800	81.350	50.175
		Std. Err.	32.626	15.232	8.424
	ESCCA	*Mean*	410.000	151.729	76.334
		Std. Err.	30.239	21.892	15.956
	ESCC	*Mean*	284.417	61.068	31.011
		Std. Err.	34.262	9.168	4.699
**ITS**	HC	*Mean*	56.077	12.456	7.845
		Std. Err.	14.490	3.756	1.902
	ESINA	*Mean*	36.900	7.905	6.517
		Std. Err.	4.037	1.460	1.344
	ESIN	*Mean*	54.900	7.576	4.947
		Std. Err.	10.814	1.933	1.204
	ESCCA	*Mean*	52.583	12.816	7.744
		Std. Err.	12.763	3.486	1.424
	ESCC	*Mean*	51.500	11.077	8.696
		Std. Err.	6.085	2.674	2.164

[a]GG2: Greengenes2 database. HOMD: Human Oral Microbiome Database. ITS: Internal Transcribed Spacer. HC: Healthy Control. ESIN: Esophageal squamous intraepithelial neoplasia. ESCC: Esophageal squamous cell carcinoma. ESINA: Esophageal squamous intraepithelial neoplasia adjacent tissues. ESCCA: Esophageal squamous cell carcinoma adjacent tissues.

Table S2-2. The *P* values of Wilcoxon test in Hill numbers (α-diversity) for different groups[a]

	Comparison	Statistics	q = 0[b]	q = 1[b]	q = 2[b]
**GG2**		≠	0.457	0.343	0.284
	HC vs. ESINA<<	<	0.790	0.172	0.142
		>	0.228	0.844	0.872
		≠	0.163	0.376	0.563
	HC vs. ESIN	<	0.928	0.828	0.739
		>	0.081	0.188	0.281
		≠	**0.000**	0.152	0.689
	HC vs. ESCCA	<	**0.000**	0.076	0.344
		>	1.000	0.932	0.675
		≠	**0.001**	0.611	0.247
	HC vs. ESCC	<	**0.000**	0.713	0.887
		>	1.000	0.306	0.124
		≠	0.280	0.075	0.105
	ESINA vs. ESIN	<	0.876	0.968	0.955
		>	0.140	**0.038**	0.053
		≠	**0.000**	0.497	0.539
	ESINA vs. ESCCA	<	**0.000**	0.248	0.752
		>	1.000	0.772	0.269
		≠	**0.004**	0.628	0.771
	ESIN vs. ESCC	<	**0.002**	0.314	0.639
		>	0.998	0.709	0.386
		≠	**0.024**	0.089	0.078
	ESCCA vs. ESCC	<	0.990	0.961	0.966
		>	**0.012**	**0.044**	**0.039**
**HOMD**		≠	1.000	0.832	0.738
	HC vs. ESINA	<	0.500	0.608	0.654
		>	0.525	0.416	0.369
		≠	0.063	0.115	0.115
	HC vs. ESIN	<	0.973	0.949	0.949
		>	**0.031**	0.058	0.058
		≠	**0.000**	0.295	0.503
	HC vs. ESCCA	<	**0.000**	0.147	0.765
		>	1.000	0.865	0.252
		≠	0.221	**0.019**	**0.026**
	HC vs. ESCC	<	0.110	0.992	0.989
		>	0.899	**0.009**	**0.013**
		≠	0.162	0.089	0.247
	ESINA vs. ESIN	<	0.930	0.962	0.891
		>	0.081	**0.045**	0.124

		≠	**0.000**	0.254	0.974
	ESINA vs. ESCCA	<	**0.000**	0.127	0.539
		>	1.000	0.886	0.487
		≠	**0.017**	0.228	0.093
	ESIN vs. ESCC	<	**0.008**	0.899	0.960
		>	0.993	0.114	0.047
		≠	**0.012**	**0.002**	**0.010**
	ESCCA vs. ESCC	<	0.995	0.999	0.996
		>	**0.006**	**0.001**	**0.005**
		≠	0.828	0.879	0.784
	HC vs. ESINA	<	0.610	0.584	0.631
		>	0.414	0.440	0.392
		≠	0.420	0.446	0.313
	HC vs. ESIN	<	0.210	0.795	0.858
		>	0.808	0.223	0.156
		≠	0.568	0.689	0.689
	HC vs. ESCCA	<	0.284	0.344	0.344
		>	0.734	0.675	0.675
		≠	0.384	0.894	1.000
	HC vs. ESCC	<	0.192	0.574	0.511
**ITS**		>	0.823	0.447	0.511
		≠	0.198	0.739	0.315
	ESINA vs. ESIN	<	0.099	0.658	0.860
		>	0.914	0.370	0.157
		≠	0.338	0.418	0.539
	ESINA vs. ESCCA	<	0.169	0.209	0.269
		>	0.847	0.810	0.752
		≠	0.817	0.418	0.283
	ESIN vs. ESCC	<	0.409	0.209	0.141
		>	0.617	0.810	0.873
		≠	0.371	0.671	0.887
	ESCCA vs. ESCC	<	0.185	0.685	0.579
		>	0.830	0.335	0.444

[a]GG2: Greengenes2 database. HOMD: Human Oral Microbiome Database. ITS: Internal Transcribed Spacer.

HC: Healthy Controls. ESIN: Esophageal squamous intraepithelial neoplasia. ESCC: Esophageal squamous cell carcinoma. ESINA: Esophageal squamous intraepithelial neoplasia adjacent tissues. ESCCA: Esophageal squamous cell carcinoma adjacent tissues.

[b]The bold values indicate statistical significance where the *P* value < 0.05.

Table S3-1. The Type–I TPLE parameters for the community spatial heterogeneity of different groups[a]

	Group	*b*-value	ln(*a*)	*R*	*P* value[b]	*n*
**GG2**	HC	2.567	1.221	0.936	**0.000**	13
	ESINA	2.275	2.006	0.931	**0.000**	10
	ESIN	2.682	0.874	0.926	**0.000**	10
	ESCCA	2.318	2.276	0.975	**0.000**	12
	ESCC	2.072	3.371	0.968	**0.000**	12
**ITS**	HC	1.618	6.088	0.890	**0.000**	13
	ESINA	1.436	6.489	0.872	**0.001**	10
	ESIN	2.574	1.375	0.966	**0.000**	10
	ESCCA	1.398	6.995	0.908	**0.000**	12
	ESCC	1.441	6.864	0.728	**0.007**	12

[a]TPLE: Taylor's Power Law Extensions. GG2: Greengenes2 database. ITS: Internal Transcribed Spacer. *b*: The *b* values correspond to the community spatial heterogeneity. ln(*a*): Parameter ln(*a*) is the intercept of the log-linear form of Taylor's Power Law. *R*: The values represent correlation coefficients. *n*: The values represent sample sizes. HC: Healthy Controls. ESIN: Esophageal squamous intraepithelial neoplasia. ESCC: Esophageal squamous cell carcinoma. ESINA: Esophageal squamous intraepithelial neoplasia adjacent tissues. ESCCA: Esophageal squamous cell carcinoma adjacent tissues.

[b]The bold values indicate statistical significance where the *P* value < 0.05.

Table S3-2. The results of the permutation tests for Type-I TPLE parameters for all pairwise comparisons[a]

	Comparsions	*b*[b]	ln(*a*)[b]
**GG2**	HC vs. ESINA	0.535	0.639
	HC vs. ESIN	0.905	0.915
	HC vs. ESCCA	0.145	0.142
	HC vs. ESCC	**0.016**	**0.010**
	ESINA vs. ESIN	0.841	0.865
	ESINA vs. ESCCA	0.790	0.658
	ESIN vs. ESCC	**0.000**	**0.001**
	ESCCA vs. ESCC	0.459	0.478
**ITS**	HC vs. ESINA	0.606	0.820
	HC vs. ESIN	**0.024**	**0.043**
	HC vs. ESCCA	0.521	0.597
	HC vs. ESCC	0.645	0.724
	ESINA vs. ESIN	**0.028**	0.075
	ESINA vs. ESCCA	0.892	0.719
	ESIN vs. ESCC	**0.039**	0.066
	ESCCA vs. ESCC	0.898	0.937

[a]TPLE: Taylor's Power Law Extensions. GG2: Greengenes2 database. ITS: Internal Transcribed Spacer. *b*: The *b* values correspond to the community spatial heterogeneity. ln(*a*): Parameter ln(*a*) is the intercept of the log-linear form of Taylor's Power Law. HC: Healthy Controls. ESIN: Esophageal squamous intraepithelial neoplasia. ESCC: Esophageal squamous cell carcinoma. ESINA: Esophageal squamous intraepithelial neoplasia adjacent tissues. ESCCA: Esophageal squamous cell carcinoma adjacent tissues.

[b]The bold values indicate statistical significance, suggesting a difference in *b*/ln(*a*) between the two groups.

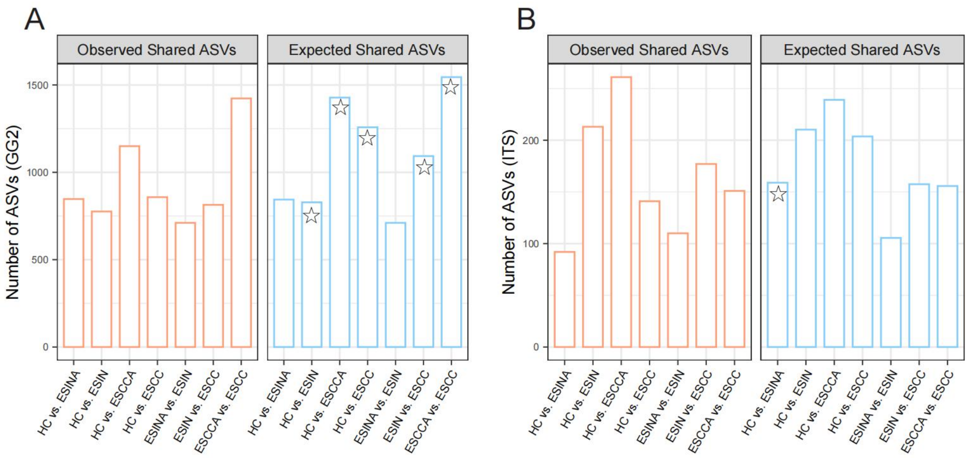

Fig. S1 The bar graphs show observed and expected shared species (*A2* algorithm) for bacterial (A) and fungal (B) comparisons across HC, ESINA, ESIN, ESCCA, and ESCC groups. The "☆" represent a statistical difference between the two cohorts in expected shared species. HC: Health controls. ESINA: Esophageal squamous intraepithelial neoplasia adjacent tissues. ESIN: Esophageal squamous intraepithelial neoplasia. ESCCA: Esophageal squamous cell carcinoma adjacent tissues. ESCC: Esophageal squamous cell carcinoma. ASVs: Amplicon sequence variants.

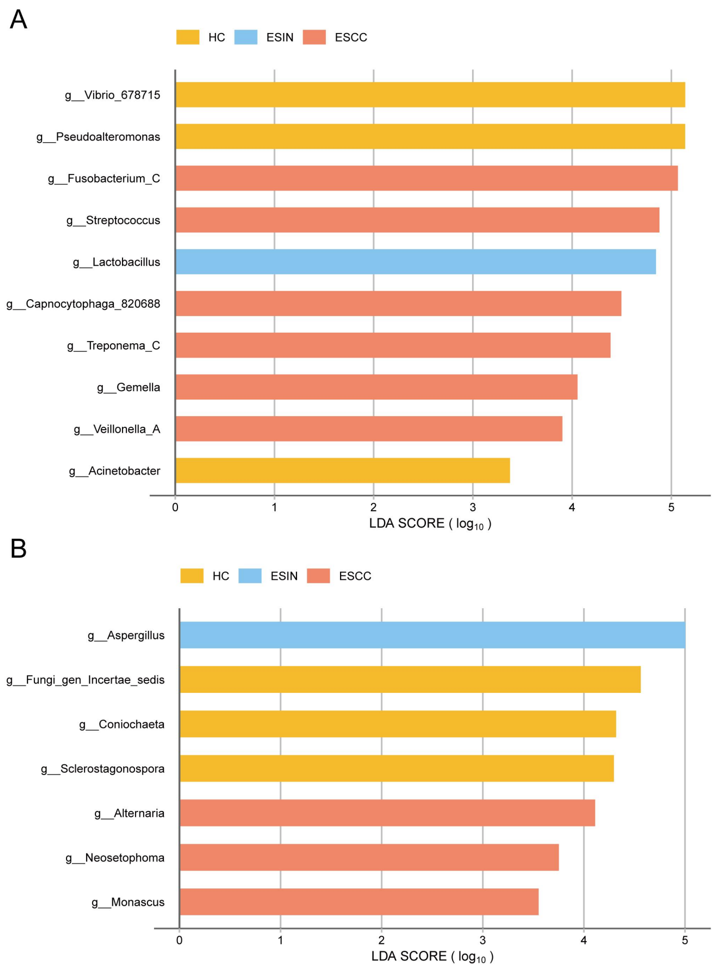

Fig. S2 LEfSe analysis showed the most abundant taxa of bacteria (A) and fungi (B) in the genus level among HC, ESIN, and ESCC groups. LEfSe: linear discriminant analysis effect size. HC: Health controls. ESIN: Esophageal squamous intraepithelial neoplasia. ESCC: Esophageal squamous cell carcinoma.

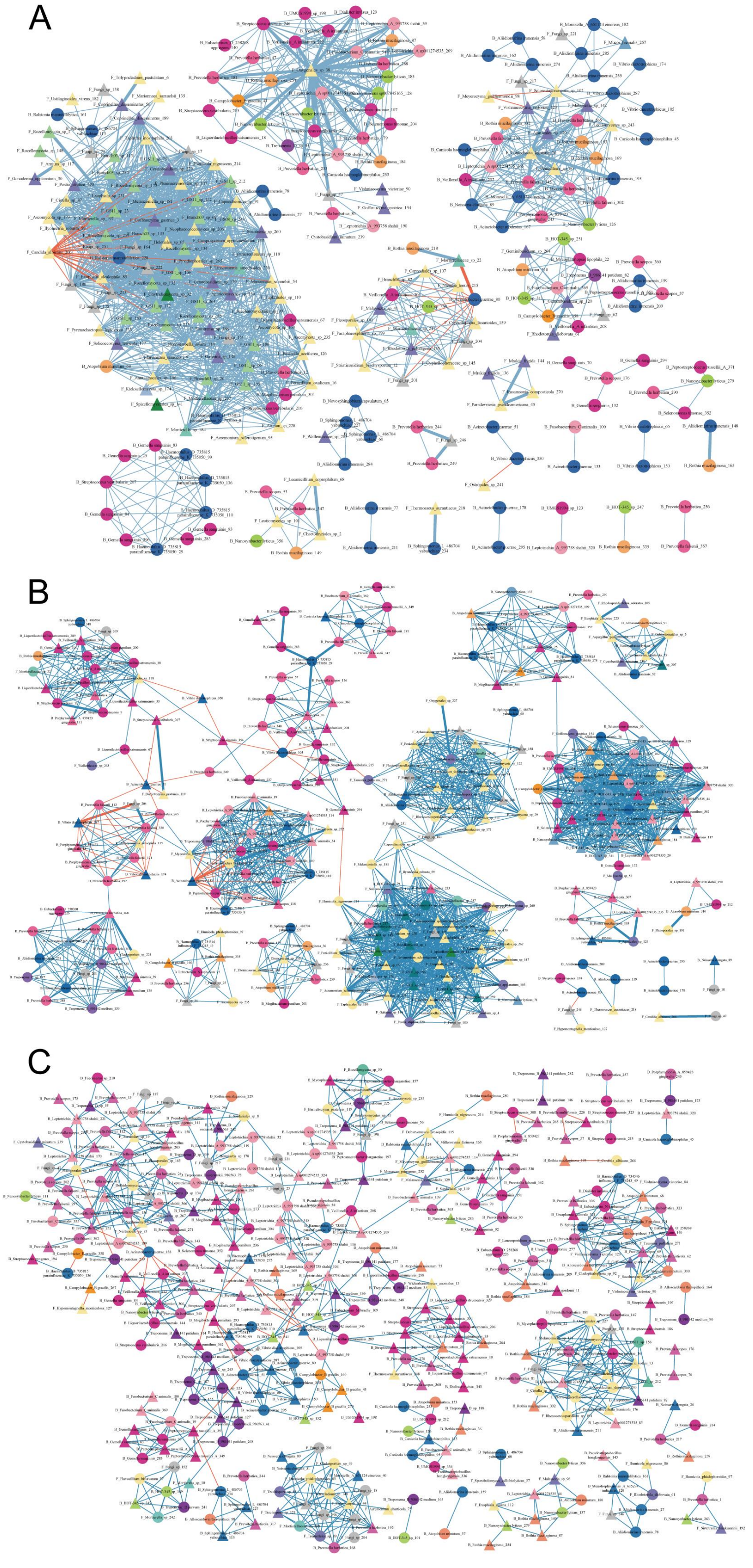

Fig. S3 The co-occurrence analysis between esophageal bacteria and fungi in HC (A), ESIN (B), and ESCC (C) groups. Circles represent core nodes; triangles indicate peripheral nodes. Each node represents a distinct species. bold edges form the backbone. The orange edges show negative correlations. blue edges show positive correlations and different colors represent microbial phyla. HC: Health controls. ESIN: Esophageal squamous intraepithelial neoplasia. ESCC: Esophageal squamous cell carcinoma.

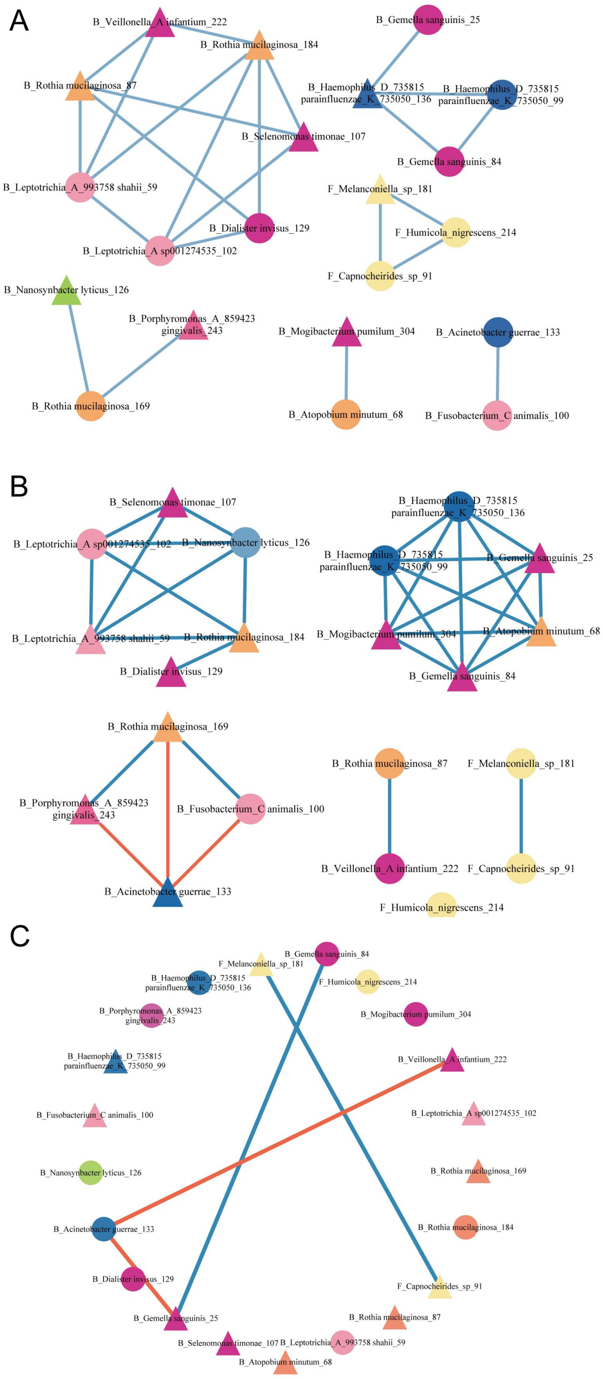

Fig. S4 The shared a bacterial-fungal co-occurrence network in HC (A), ESIN (B), and ESCC (C) groups. Circles represent core nodes. triangles indicate peripheral nodes. Each node represents a distinct species. bold edges form the backbone. Orange edges show negative correlations. blue edges show positive correlations and different colors represent microbial phyla. HC: Health controls. ESIN: Esophageal squamous intraepithelial neoplasia. ESCC: Esophageal squamous cell carcinoma.
