# Supplementary material for: Esophageal cancer and precancerous lesions: focus on resident bacteria and fungi
Source: Microbiol Spectr. 2025 May 20;13(7):e03137-24. doi: 10.1128/spectrum.03137-24 (PMC12210859; doi:10.1128/spectrum.03137-24)
Supplement: Supplemental data — Sampling workflow and quality control measures. [file spectrum.03137-24-s0002.pdf]

Online Supplementary Information (OSI) for: Esophageal Cancer and Precancerous Lesions: Focus on Resident Bacteria and Fungi

List of Online Supplementary materials:

The first page is sampling workflow. ESD: endoscopic submucosal dissection. HC: healthy control. ESINA: esophageal squamous intraepithelial neoplasia adjacent tissue. ESIN: esophageal squamous intraepithelial neoplasia. ESCCA: esophageal squamous cell carcinoma adjacent tissues. ESCC: esophageal squamous cell carcinoma.

The second page is quality control measures.

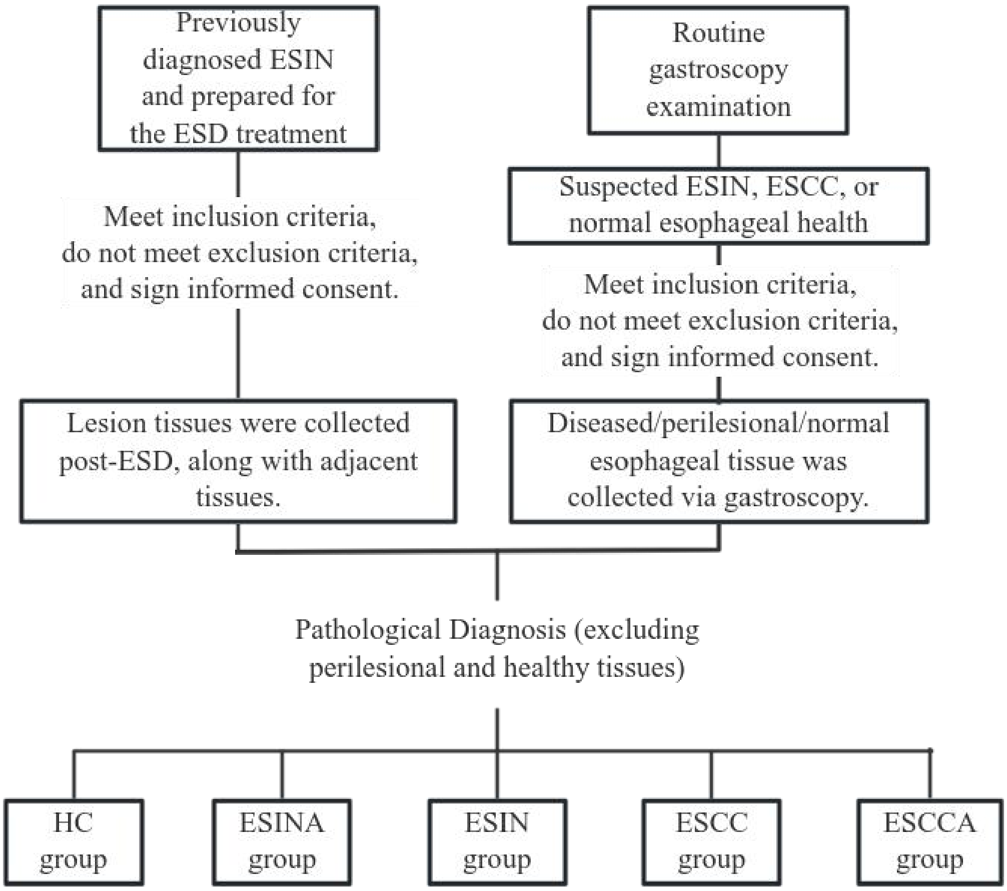

Sampling workflow ESD: endoscopic submucosal dissection. HC: healthy control. ESINA: esophageal squamous intraepithelial neoplasia adjacent tissue. ESIN: esophageal squamous intraepithelial neoplasia. ESCCA: esophageal squamous cell carcinoma adjacent tissues. ESCC: esophageal squamous cell carcinoma.

Quality control measures

The quality control team consisted of three experts, each with over five years of experience in gastroscopy, specifically focused on early gastrointestinal cancer detection. A consensus between two experts was required for approval; if there was a disagreement, the third expert made the final decision. Quality control was categorized into three aspects: aseptic technique during sampling, control over specimen size and location, and final pathology quality.

For aseptic technique during sampling: 1. Biopsy of the esophageal tissue was performed from the anal side toward the oral side. 2. Prior to sampling, 5-10 ml of sterile saline was used to flush the biopsy channel and the esophageal tissue. 3. When sampling different tissue areas, an additional flush of 5-10 ml of sterile saline was administered to the biopsy channel, esophageal tissue, and biopsy forceps. 4. After sampling, the tissue was promptly transferred with a sterile cotton swab to a cryovial and quickly froze into liquid nitrogen.

The tissue sample size was approximately 1 × 2 mm, with 1-2 samples obtained. For the ESIN and ESCC groups, biopsies were taken from the center of the lesion. For the ESINA/ESCCA group, biopsies were obtained from the esophageal tissue located more than 5 cm from the ESIN/ESCC lesion. In the HC group, tissue samples were taken from the esophageal tissue approximately 25 cm from the incisor teeth.

Grouping of patients was determined based on the final pathological outcome.
